# Downregulated lncRNA GAS5 and Upregulated miR-21 Lead to Epithelial–Mesenchymal Transition and Lung Metastasis of Osteosarcomas

**DOI:** 10.3389/fcell.2021.707693

**Published:** 2021-07-27

**Authors:** Ying Wang, Xue Ren, Ye Yuan, Bao-Shan Yuan

**Affiliations:** ^1^Department of Medicine Laboratory, The First Hospital of Jilin University, Changchun, China; ^2^Department of Oncological Gynecology, The First Hospital of Jilin University, Changchun, China

**Keywords:** osteosarcoma, lung metastasis, Gas5, miR-21, EMT

## Abstract

Lung is the primary site of osteosarcoma metastasis, but the underlying genetic or epigenetic factors determining lung metastasis of osteosarcoma are unknown. In this study, we report the status of growth arrest specific 5 (GAS5) in lung metastatic osteosarcomas. GAS5 was generally downregulated in osteosarcoma patients (*n* = 24) compared to healthy controls (*n* = 10) and even more so in patients with lung metastatic disease(*n* = 11) compared to the patients without metastasis (*n* = 13). We also report a role of miR-21 in GAS5-mediated effects. Downregulation of GAS5 in hFOB 1.19 and U2OS osteosarcoma cells enhanced their migration and invasion, along with an upregulated epithelial–mesenchymal transition (EMT), as evidenced by downregulated E-cadherin and upregulated vimentin, ZEB1, and ZEB2. Downregulation of GAS5 also resulted in a significantly increased expression of miR-21. Moreover, downregulation of such elevated miR-21 was found to reverse the effects of GAS5 silencing. miR-21 was also found to be elevated in osteosarcoma patients with its levels particularly high in patients with lung metastasis. Our observations reveal a possible role of GAS5 and miR-21 in lung metastasis of osteosarcoma, presenting them as novel targets for therapy.

## Introduction

Osteosarcoma is a type of bone cancer diagnosed in young adults and teenagers more frequently than at a later stage (Wittig et al., 2002). It is more common in long bones such as those in the legs (Ferguson and Turner, 2018) but can be diagnosed in any bone. By some estimates, osteosarcomas account for almost one-fifth of all bone cancers (Kundu, 2014). Also referred to as osteogenic sarcoma, this cancer is known to metastasize to patients’ lungs (Marcove et al., 1970; Zhang J. et al., 2020), which often is the cause of associated mortality. Lungs are one of the most common sites of cancer metastasis (Jamil and Kasi, 2021) including from osteosarcomas (Saha et al., 2013), and the mechanisms and underlying causes of lung metastases of primary osteosarcomas largely remain unknown. Moreover, the 5-year survival rates of osteosarcoma patients with lung metastatic disease are quite dismal (Farfalli et al., 2015), and thus, there is an urgent need to better understand the genetic or the epigenetic factors that can regulate lung metastasis of osteosarcomas.

Recently, a long non-coding RNA (lncRNA) MALAT1 was reported to be upregulated in lung metastatic osteosarcomas (Zhang J. et al., 2020). Not only was this oncogenic lncRNA found elevated in lung metastases of osteosarcoma patients, but also its levels were generally high in various osteosarcoma cell lines, particularly in the lung-metastatic derivatives. This lncRNA sponged miR-202 as a mechanism for its involvement in the lung metastasis of osteosarcoma (Zhang J. et al., 2020). In general, there has been a lot of interest in the last few years in the lncRNAs in osteosarcoma (Li et al., 2016; Zhang Y. et al., 2020; Ghafouri-Fard et al., 2021), and they have been proposed as legitimate biomarkers as well as targets for therapy in osteosarcomas (Li et al., 2017). They are being investigated for their regulation of drug resistance mechanisms in osteosarcoma (Xu et al., 2020; Ferretti and León, 2021) as well the metastasis of osteosarcomas (Yang et al., 2016; Zhang J. et al., 2020). The interactions of lncRNAs with miRNAs in osteosarcoma are also being investigated (Wang J. Y. et al., 2020; Zhang J. et al., 2020), with one particular miRNA, the miR-21, emerging as a miRNA of interest in osteosarcomas (Sekar et al., 2019).

One lncRNA that has been investigated in a number of reports on osteosarcoma is the lncRNA growth arrest specific 5 (GAS5). This is a tumor suppressor lncRNA generally reported to be downregulated in human cancers (Yu and Hann, 2019). In osteosarcoma cells, dysregulated levels of GAS5 can impact cell growth and proliferation (Wang and Kong, 2018; Liu et al., 2020). GAS5 can predict cancer metastasis (Song et al., 2016). It is frequently downregulated in metastatic cancers (Wu et al., 2016). However, GAS5 has not been evaluated for its potential role in metastasis of osteosarcomas, particularly the lung metastasis of osteosarcomas, which prompted us to first evaluate this lncRNA in patient samples and then explore the underlying mechanisms.

## Materials and Methods

### Patients

All the osteosarcoma patients as well as healthy control individuals were enrolled at Jilin University Hospital in Changchun, and informed consent was obtained. The study was reviewed and approved by the Ethics Committee at the Jilin University (Approval Number 20/756). Diagnosis of osteosarcoma was the primary inclusion criteria. In patients with metastatic disease, diagnosis of lung metastasis was the inclusion criteria. The patient demographics are provided in Table 1. All tissue biopsies were stored at −80°C until they were being analyzed.

**TABLE 1:** Patient Demographics.

**Group**		**N**	**Age range (years)**	**Mean age (years)**	**Males (%)**	**Females (%)**
Control	10	12–45	19.8	6 (60.00)	4 (40.00)	
Osteosarcoma	No metastasis	13	12–44	20.8	8 (61.54)	5 (38.46)
	Lung Metastasis	11	13–42	21.2	7 (63.64)	4 (36.36)

### Cell Lines

Osteosarcoma cells KRIB, SaOS, MG63, and U2OS were purchased from American Type Culture Collection (ATCC, United States). All of these cell lines were grown in filter-sterilized Dulbecco’s modified Eagle’s medium (DMEM) with 10% fetal bovine serum at 37°C. hFOB 1.19 cells were also purchased from ATCC (United States) and grown in F12-DMEM with 10% fetal bovine serum and 0.3 mg/ml G418. All of the cell lines were cultured in tissue culture incubators with 5% CO_2_.

### Migration and Invasion Assays

For evaluation of migration and invasion potential, cells were trypsinized, collected, resuspended in serum-free medium, and counted. Cells (1 × 10^5^) were seeded into a non-coated (for migration assay) or Matrigel (for invasion assay)-coated (BD Bioscience, China) chamber (Corning, China), and the chamber was placed on a well containing normal culture media with 10% serum. After 20 h of growth, cells still in the Matrigel were removed using a cotton swab, and the cells that had invaded through the Matrigel and were now on the lower membrane surface were fixed with 4% paraformaldehyde and stained with 0.1% crystal violet. Cells were then counted under a bright field microscope by two independent personnel.

### RNA Extraction and Real-Time PCR

Total RNA from osteosarcoma cells was extracted using TRIzol reagent (Sigma, China). For GAS5 analysis, RNA was reverse transcribed to complementary DNA (cDNA) using PrimeScript RT Master Mix (TaKaRa, Japan), followed by quantitative analysis using SYBR Premix Ex Taq (TaKaRa, China). For miR-21 analysis, RNA was reverse transcribed into cDNA using miRNA-specific primers and a TaqMan MicroRNA Reverse Transcription Kit (Applied Biosystems, China). Then, cDNA was amplified using TaqMan Universal PCR Master Mix. U6 was used as an endogenous control. 2^–Δ^ ^Δ^ ^*Ct*^ calculations were used for gene quantitation.

### si-GAS5, Anti-miR-21, and Transfections

Specific small-interfering RNA (siRNA) against GAS5 and the anti-miR-21 oligos were purchased from Thermo Fisher Scientific (China). Anti-miR-21s were transfected at concentrations of 15 nM in the target cells, using DharmaFect reagent (Dharmacon, China), following standard procedures with mixing of siRNA and transfection with serum-free media before mixing them all together and replacing the normal media on cells with the mix. Transfections were performed when the cells were 50–60% confluent, and transfected cells were left for 48 h post-transfection before use in the individual experiments.

### Statistical Analyses

Data were analyzed by a trained biostatistician. *p* values were calculated using Student’s *t*-test, one-way ANOVA, and Pearson’s correlation analysis. Representative results from three repeats were presented. For our analysis, we only considered *p* < 0.05 to be statistically significant.

## Results

### Downregulation of GAS5 in Osteosarcoma Patients and Cell Lines

**FIGURE 1:**
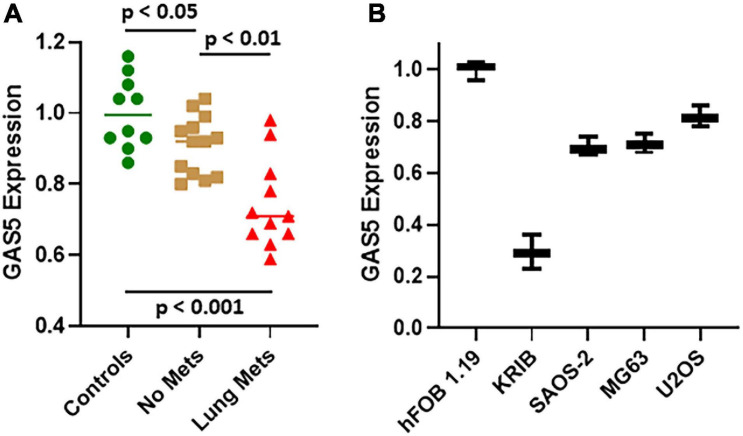
Growth arrest specific 5 (GAS5) expression in osteosarcoma patients and cell lines. Expression of GAS5 was analyzed in panel **(A)** patient samples and **(B)** cell lines, using quantitative real-time PCR (qRT-PCR). Individual *p* values are mentioned for the direct comparisons of individual patient groups.

We first evaluated the levels of GAS5, by quantitative real-time PCR (qRT-PCR), in osteosarcoma patients (*n* = 24) and compared the levels of GAS5 in patients with those in the archived samples from healthy controls (*n* = 10). Of the 24 osteosarcoma patients, 11 patients were those with lung metastatic disease, while 13 were never diagnosed with any metastasis. Our analysis revealed that compared to controls, GAS5 was significantly downregulated even in the osteosarcoma patients without metastasis (Figure 1A) (*p* < 0.05). The downregulation of GAS5 was even more significant in the osteosarcoma patients with lung metastasis (*p* < 0.001 vs. controls and *p* < 0.01 vs. osteosarcoma patients with no metastasis) (Figure 1A). Our next goal was to work out the mechanism of action of GAS5 in osteosarcoma, and therefore, we turned to cell line models for detailed mechanistic studies. Our analysis of GAS5 levels in different osteosarcoma cell lines revealed that in hFOB 1.19, the normal control cells had the highest expression of GAS5 (Figure 1B). All the osteosarcoma cell lines had relatively downregulated GAS5 (Figure 1B) confirming the downregulation of GAS5 in our cell line models as well. hFOB 1.19 cells have been well characterized as immortalized, but non-transformed osteoblastic cells (Subramaniam et al., 2002).

### Implication of Downregulating GAS5 in Osteosarcoma Cells

**FIGURE 2:**
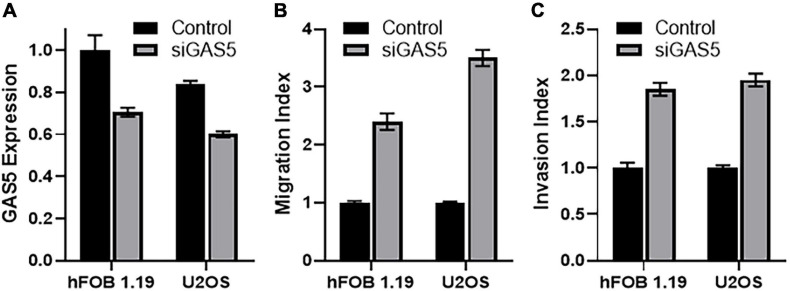
Downregulation of growth arrest specific 5 (GAS5) in osteosarcoma cells affects migration and invasion of cells. **(A)** GAS5 was silenced by siGAS5 in hFOB 1.19 and U2OS cells, and the resulting effect on **(B)** migration and **(C)** invasion was analyzed. Control cells (without GAS5 silencing) were assigned a value of “1,” and the corresponding differences in GAS5-silenced cells are shown. Under all conditions, *p* < 0.01.

We chose hFOB 1.19 and U2OS cells for further mechanistic studies, as these cells were found to be the ones with highest endogenous GAS5 levels, and we wanted to study the implications of downregulating GAS5 levels as would normally happen in tumor development and/or cancer metastasis. The siRNA against GAS5 resulted in the efficient downregulation of GAS5 levels (Figure 2A). Moreover, the downregulation of GAS5 was significant (*p* < 0.01) in both the cell lines, hFOB 1.19 and U2OS. Next, we checked the migration and invasion potential of hFOB 1.19 and U2OS cells with and without silencing of GAS5. Compared to control cells, the cells, both hFOB 1.19 and U2OS, with downregulated GAS5 had significantly increased migration (Figure 2B) (*p* < 0.01) and invasion index (Figure 2C) (*p* < 0.01).

### Downregulation of GAS5 Affects EMT

**FIGURE 3:**
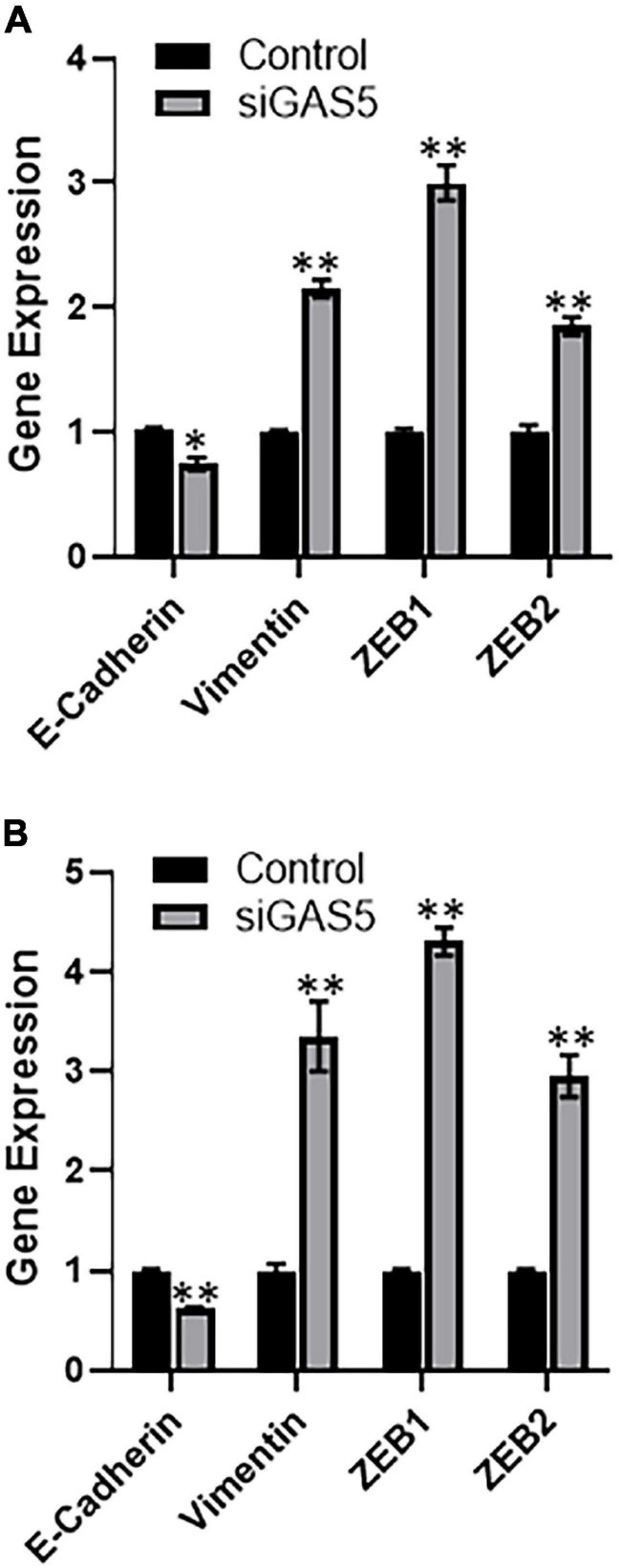
Downregulation of growth arrest specific 5 (GAS5) induces epithelial–mesenchymal transition (EMT). GAS5 was downregulated by small-interfering RNA (siRNA) in panel **(A)** hFOB 1.19 and **(B)** U2OS cells, and the resulting effects on the expression of EMT genes, as indicated, were analyzed. Control cells (without GAS5 silencing) were assigned a value of “1,” and the corresponding differences in GAS5-silenced cells are shown. glyceraldehyde 3-phosphate dehydrogenase (GAPDH) was used as the internal control for analysis. *p < 0.05 and **p < 0.01.

Growth arrest specific 5 has been reported to affect the process of EMT (Liu et al., 2018), including in osteosarcomas (Ye et al., 2017). Therefore, we next investigated the effect of GAS5 downregulation on EMT in our cell line models. When GAS5 was silenced in hFOB 1.19 cells, the epithelial marker E-cadherin was significantly downregulated (*p* < 0.05), while mesenchymal markers vimentin, ZEB1, and ZEB2 were significantly upregulated (Figure 3A) (*p* < 0.01). In the U2OS cells as well, our analysis revealed similar results, and we observed significant downregulation of epithelial marker and significant upregulation of mesenchymal markers (Figure 3B) (*p* < 0.01). All of these results point to reciprocal relationship between GAS5 expression and EMT and, therefore, downregulation of GAS5 results in induction of EMT.

### GAS5 Sponges miR-21

**FIGURE 4:**
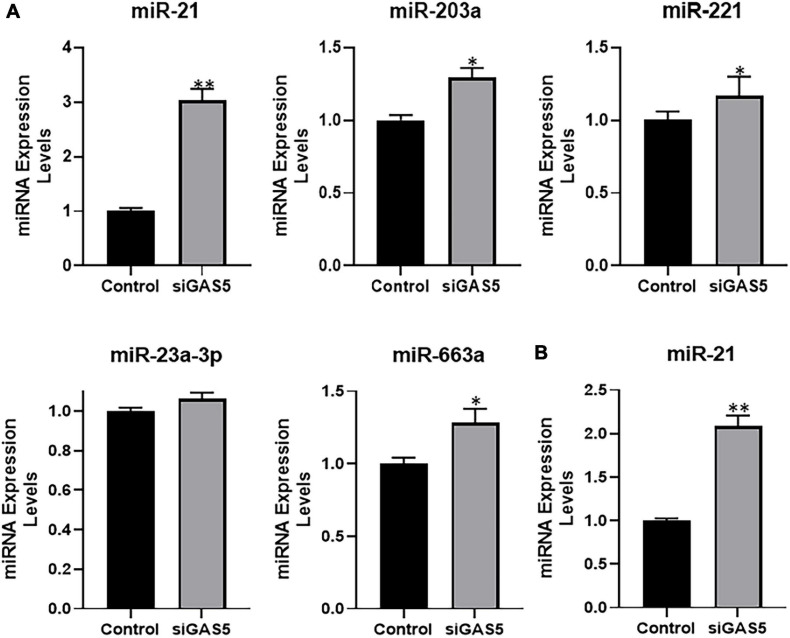
Growth arrest specific 5 (GAS5) sponges miR-21. **(A)** GAS5 was downregulated by small-interfering RNA (siRNA) in U2OS cells, and the resulting effects on the expression of different miRNAs were analyzed. **(B)** GAS5 was downregulated by siRNA in hFOB 1.19 cells, and the resulting effect on the expression of miR-21 was analyzed. Control cells (without GAS5 silencing) were assigned a value of “1,” and the corresponding differences in GAS5-silenced cells are shown. *p < 0.05 and **p < 0.01.

Next, we evaluated the expression levels of several miRNAs in GAS5-silenced U2OS cells in an attempt to find the miRNA that is sponged by GAS5. We checked the expression levels of several miRNAs that have been reported in the literature to be sponged by GAS5 in addition to some novel ones. Several miRNAs, such as miR-203a, miR-221, and miR-663a, were found to be significantly upregulated in GAS5-silenced cells (Figure 4A) (*p* < 0.05). However, miR-21 was observed to be the most upregulated miRNA in GAS5-silenced U2OS cells with its levels ∼3-fold higher in silenced cells (Figure 4A) (*p* < 0.01). We further confirmed our findings in hFOB 1.19 cells and found that silencing of GAS5 led to significantly increased expression of miR-21 in hFOB 1.19 cells as well (Figure 4B) (*p* < 0.01).

### miR-21 Reverses GAS5-Silencing Effects

**FIGURE 5:**
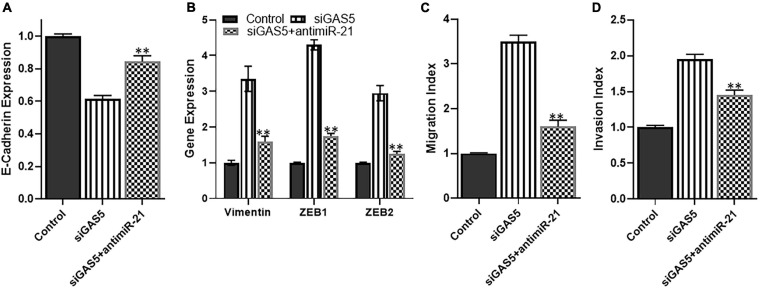
miR-21 reverses growth arrest specific 5 (GAS5)-silencing effects. The effect of microRNA (miRNA) miR-21 to reverse the effects GAS5 silencing was studied in U2OS cells. GAS5 was downregulated by siRNA in U2OS cells, followed by transfections of anti-miR-21. The resulting effects on panel **(A)** expression of epithelial marker E-cadherin; **(B)** expression of mesenchymal markers vimentin, ZEB, and ZEB2; **(C)** migration index; and **(D)** invasion index were analyzed. Glyceraldehyde 3-phosphate dehydrogenase (GAPDH) was used as the internal control for gene expression analysis. **p < 0.01.

Since miR-21 was observed in our analysis to be the most upregulated miRNA upon GAS5 silencing, we next analyzed whether downregulating this miRNA can attenuate the effects of GAS5 silencing. We used anti-miR-21s to downregulate miR-21 and observed that downregulating miR-21 in GAS5-silenced U2OS cells brought the levels of epithelial marker E-cadherin up significantly (Figure 5A) (*p* < 0.01). It also brought down the levels of mesenchymal markers significantly (Figure 5B) (*p* < 0.01). These results suggest that miR-21 plays a role in EMT induction by GAS5. In addition to the effects on EMT induction, we also checked the migration and potential index when miR-21 was downregulated in GAS5-silenced cells. Our analysis revealed that such downregulation of miR-21 in GAS5-silenced U2OS cells resulted in bringing down the migration and invasion of U2OS, almost to normal levels (Figures 5C,D) (*p* < 0.01).

### miR-21 in Lung Metastases of Osteosarcoma

**FIGURE 6:**
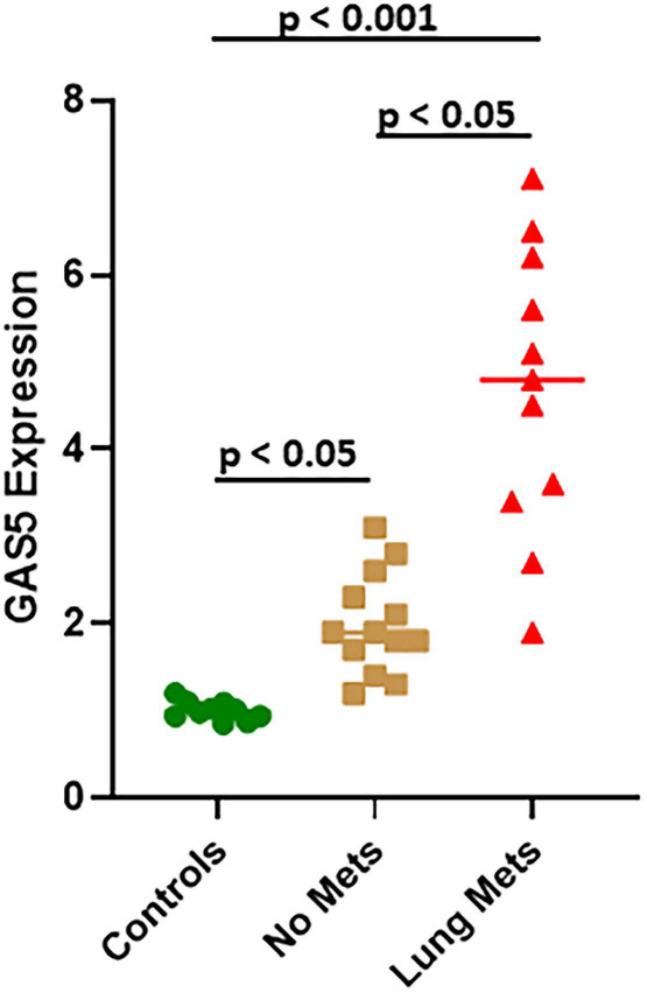
miR-21 in lung metastases of osteosarcoma. Expression of miR-21 was analyzed in osteosarcoma patient samples using quantitative real-time PCR (qRT-PCR). Individual *p* values are mentioned for the direct comparisons of individual patient groups.

miR-21, as presented above, is involved in GAS5 effects on osteosarcoma cells, and its upregulation can reverse the effects of GAS5 silencing. Therefore, we next explored if miR-21 levels correlate inversely with those of GAS5 in patients with lung metastases as well. Our analysis revealed that miR-21 levels are significantly upregulated in osteosarcoma patients (even those without metastases), compared to healthy controls (Figure 6) (*p* < 0.05). The upregulation of miR-21 was even more significant (*p* < 0.001) in osteosarcoma patients with lung metastases, and interestingly, patients with lung metastases had significantly elevated levels of miR-21 compared to those with no metastases (Figure 6) (*p* < 0.05).

## Discussion

An interesting aspect about osteosarcoma that makes it unique is that osteosarcoma is more frequently diagnosed at an early age, as compared to later stage. In Chinese population, the average age at diagnosis is ∼21 years, and the male individuals are more frequently diagnosed, ∼1.7-fold higher, than female (Wang et al., 2017). The peak age of osteosarcoma diagnosis is between the ages of 10 and 20 (Wang et al., 2017). This is in clear contrast to several other cancers that correlate positively with old age. Our analyses involved evaluation of lncRNA and its sponged miRNA in patient samples, in addition of elucidation of mechanism in cell lines. The analysis of patient samples increases confidence in our observations. Our patient cohort consisted of 13 osteosarcoma patients with no metastasis and 11 osteosarcoma patients with lung metastasis. Comparing these two distinct groups of osteosarcoma patients helped us identify the signature associated with the lung-specific metastasis.

In recent years, there has been a lot of interest in the lncRNAs in human cancers (Rasool et al., 2016; Balas and Johnson, 2018; Carlevaro-Fita et al., 2020). Even in osteosarcomas, a diagnostic and prognostic importance of these non-coding RNAs has been recognized. The lung metastasis of primary osteosarcoma is an understudied topic with regards to a possible role of lncRNAs, and the only available report is the one that described a possible involvement of lncRNA MALAT1 (Zhang J. et al., 2020). There are many similarities and a few differences in our observations compared to that study. Similar to our analyses, the published report on MALAT1 also evaluated lncRNA expression in patient samples. However, the lncRNA MALAT1 is oncogenic in nature and was found to be upregulated in patients with lung metastasis. In contrast, the GAS5 lncRNA evaluated by us is a tumor suppressor. Accordingly, the levels of GAS5 were significantly downregulated in patients with lung metastasis. In the report on MALAT1, miR-202 was reported to be the miRNA sponged by oncogenic MALAT1. This means that miR-202 should be a tumor suppressor, and such tumor suppressor role of miR-202 has been reported in the literature (Wang J. et al., 2020). On the other hand, our analysis revealed miR-21 to be miRNA sponged by GAS5. Given the tumor-suppressive nature of GAS5, miR-21 should be oncogenic, and reports on miR-21 confirm such oncogenic role of this miRNA (Javanmardi et al., 2017; Najjary et al., 2020).

In our analysis for the role of GAS5 in osteosarcoma cells metastasis, we performed migration and invasion assays. These are assays for the assessment of the metastasis potential of cancer cells *in vitro*. Our analysis revealed that both migration and invasion of osteosarcoma cells increased when GAS5 was downregulated. These observations further confirm the tumor-suppressive nature of GAS5 and validate our findings in patient samples where GAS5 was found to be downregulated. As a mechanism, we validated EMT as the underlying cause of metastasis. The relationship between GAS5 and EMT is not new, and there are several reports on the subject (Ye et al., 2017; Liu et al., 2018; Yu and Hann, 2019). However, we present novel evidence for the possible role of EMT in lung metastasis of osteosarcoma. Targeting EMT could be a novel approach to limit or treat lung metastasis of osteosarcomas.

In our analysis, we observed sponging of miR-21 by GAS5. Such sponging of miR-21 was found to be much more than all the miRNAs tested. Some of the tested miRNAs have previously been shown to be targeted by GAS5 in osteosarcoma, such as miR-221 (Ye et al., 2017), miR-203a (Wang and Kong, 2018), miR-23a (Liu et al., 2020), and miR-663a (Yao et al., 2020; Zhao et al., 2020). Our analysis thus yielded novel information on the miRNA that could be targeted by GAS5. Interestingly, a role of miR-21 in osteosarcoma has been suggested (Sekar et al., 2019), and our observations bring the attention again to this miRNA for further evaluations. According to our observations, more likely miR-21 is regulated by GAS5. This is in accordance with the general sponging of miRNAs by lncRNAs. However, there is some evidence suggesting that GAS5 can itself be regulated by miR-21 (Zhang et al., 2013). This is an interesting revelation and suggests that the regulatory relationship between GAS5 and miR-21 could be mutual. There might even be a reciprocal relationship that needs to be further explored, particularly in reference to how this relationship and counter-regulation might affect lung metastasis of osteosarcomas.

In our study, we determined a role of miR-21 in EMT of osteosarcoma, particularly in the context of regulation by lncRNA GAS5. EMT regulation by miR-21 has been suggested in some earlier studies as well (Bornachea et al., 2012; Dai et al., 2019), and such miR-21-regulated EMT has been linked to cancer metastasis (Xiao and Jie, 2019). However, we present here some very novel information for a role of miR-21 in GAS5-regulated lung metastases of osteosarcomas with underlying effect on the process of EMT. Another interesting revelation from our analysis was that we observed a certain degree of specificity in terms of a role of GAS5 and miR-21 in lung metastasis of osteosarcomas. We make this comment because not only GAS5 was downregulated in osteosarcomas in general, it was significantly further reduced in lung metastases. Similarly, we observed upregulated miR-21 in osteosarcoma patients, even those without metastasis, thus indicating a role of miR-21 in osteosarcoma. However, the levels of miR-21 were significantly upregulated in osteosarcoma patients with lung metastasis, compared to those with no metastasis. These results seem to suggest a possible specific role of GAS5-miR-21 in lung metastasis of osteosarcoma. In our study, we did not study GAS5 levels in metastasis of osteosarcoma to other organs, and such studies should further help determine any specificity of GAS5-miR-21 in tissue specific metastasis.

## Data Availability Statement

The original contributions presented in the study are included in the article/supplementary material, further inquiries can be directed to the corresponding author.

## Ethics Statement

The studies involving human participants were reviewed and approved by Ethics Committee at the Jilin University (Approval Number 20/756). Written informed consent to participate in this study was provided by the participants’ legal guardian/next of kin.

## Author Contributions

XR and B-SY performed the experiments and collected the data. XR, YY, and YW analyzed the results and prepared the figures. YY drafted the manuscript and gathered the resources. All authors edited the manuscript and approved the final version.

## Conflict of Interest

The authors declare that the research was conducted in the absence of any commercial or financial relationships that could be construed as a potential conflict of interest.

## Publisher’s Note

All claims expressed in this article are solely those of the authors and do not necessarily represent those of their affiliated organizations, or those of the publisher, the editors and the reviewers. Any product that may be evaluated in this article, or claim that may be made by its manufacturer, is not guaranteed or endorsed by the publisher.
